# Age-Associated mRNA and miRNA Expression Changes in the Blood-Brain Barrier

**DOI:** 10.3390/ijms20123097

**Published:** 2019-06-25

**Authors:** Emily F. Goodall, Vicki Leach, Chunfang Wang, Johnathan Cooper-Knock, Paul R. Heath, David Baker, David R. Drew, M. Jill Saffrey, Julie E. Simpson, Ignacio A. Romero, Stephen B. Wharton

**Affiliations:** 1 Sheffield Institute for Translational Neuroscience, 385a Glossop Road, University of Sheffield, Sheffield S10 2HQ, UK; e.goodall@sheffield.ac.uk (E.F.G.); vleach1@sheffield.ac.uk (V.L.); j.cooper-knock@sheffield.ac.uk (J.C.-K.); p.heath@sheffield.ac.uk (P.R.H.); d_baker@live.com (D.B.); d.drew@sheffield.ac.uk (D.R.D.); s.wharton@sheffield.ac.uk (S.B.W.); 2 School of Life Science, Health and Chemical Sciences, Faculty of Science, Technology Engineering and Mathematics, The Open University, Walton Hall, Milton Keynes MK7 6AA, UK; cjoyce.wang@gmail.com (C.W.); jill.saffrey@open.ac.uk (M.J.S.); nacho.romero@open.ac.uk (I.A.R.)

**Keywords:** ageing, blood brain barrier, gene expression, miRNA

## Abstract

Functional and structural age-associated changes in the blood-brain barrier (BBB) may affect the neurovascular unit and contribute to the onset and progression of age-associated neurodegenerative pathologies, including Alzheimer’s disease. The current study interrogated the RNA profile of the BBB in an ageing human autopsy brain cohort and an ageing mouse model using combined laser capture microdissection and expression profiling. Only 12 overlapping genes were altered in the same direction in the BBB of both ageing human and mouse cohorts. These included genes with roles in regulating vascular tone, tight junction protein expression and cell adhesion, all processes prone to dysregulation with advancing age. Integrated mRNA and miRNA network and pathway enrichment analysis of the datasets identified 15 overlapping miRNAs that showed altered expression. In addition to targeting genes related to DNA binding and/or autophagy, many of the miRNAs identified play a role in age-relevant processes, including BBB dysfunction and regulating the neuroinflammatory response. Future studies have the potential to develop targeted therapeutic approaches against these candidates to prevent vascular dysfunction in the ageing brain.

## 1. Introduction

The blood-brain barrier (BBB), formed by capillary endothelial cells, the basement membrane and surrounding pericytes and astrocyte endfeet, is a highly specialised structure that maintains homeostasis within the central nervous system (CNS) by regulating the bidirectional flow of molecules between the circulation and the brain parenchyma [1]. Functional and structural age-associated BBB changes may affect the neurovascular unit (NVU), impacting vascular integrity and resulting in alterations in the perivascular environment, neuronal function and the neuroinflammatory response, as demonstrated in both human and mouse studies [2,3,4].

BBB dysfunction, a reduction in cerebral blood flow and impaired haemodynamic responses are prominent features of a range of neurodegenerative diseases, including Alzheimer’s disease (AD) [5,6]. In addition to the findings of several post-mortem tissue studies [7,8,9], neuroimaging approaches have demonstrated that dysfunction of the BBB is a feature of ageing [10], mild cognitive impairment and early AD [11,12]. Moreover, increased permeability of the BBB precedes the formation of senile plaque formation in an animal model of AD [13]. Together, these data support the vascular hypothesis of AD [14,15,16], and suggest that BBB dysfunction, which can occur before the clinical manifestation of dementia, is a prospective therapeutic target.

The continued advancement of transcriptomic profiling techniques, including microarray analysis, has enabled the identification of specific gene expression changes and biological processes associated with ageing [17,18,19,20,21]. Laser capture microdissection (LCM) enables the isolation of regions or enrichment of specific cell types from post-mortem material and has been used in conjunction with transcriptomic analysis in both human and animal model ageing studies to identify differentially expressed genes in the hippocampus [22], and in enriched populations of LCM-isolated neurones [23,24,25] and astrocytes [26]. While LCM has been used to selectively study the transcriptomic profile of LCM-isolated vessels in neurocysticercosis [27,28], glioblastoma [29,30], multiple sclerosis [31] and schizophrenia [32], to date no studies have employed this approach to identify gene expression changes associated with normal ageing.

MicroRNAs (miRNAs) are small, non-coding RNA molecules (containing approximately 22 nucleotides) that target genes in a sequence-specific manner to modulate gene expression, mainly by degradation of mRNA or repression of expression [33,34]. They have recently been shown to target genes which regulate BBB permeability in animal models of ischaemic stroke [35] and human brain microvascular endothelial cells in vitro [36], indicating a role for miRNAs in modifying the integrity of the BBB.

Our previous histological characterisation of ageing human and mouse cohorts provides evidence of BBB dysfunction, loss of pericyte coverage and astrogliosis in normal human and murine brain ageing [37]. The current study extends these findings and investigates age-associated changes in the transcriptomic and miRNA profile of the NVU in ageing human and mouse cohorts, with the aim of defining age-associated gene expression changes and identifying potential targets to improve healthy brain ageing.

## 2. Results

### 2.1. Age-Associated Gene Expression Changes in the BBB

Gene expression analyses are sensitive to the presence of sample outliers, therefore rigorous quality control procedures were used to ensure the highest possible level of quality for both the human and mouse microarray datasets. Three human and two mouse arrays failed the quality control protocols due to a low number of present calls and were excluded from the analysis. The data was re-analysed after these cases were removed. All significant age-associated mRNA expression changes in the BBB of humans and mice are freely available at Gene Expression Omnibus (human array data accession number GSE127710 and mouse array data accession number GSE127709).

**Figure 1:**
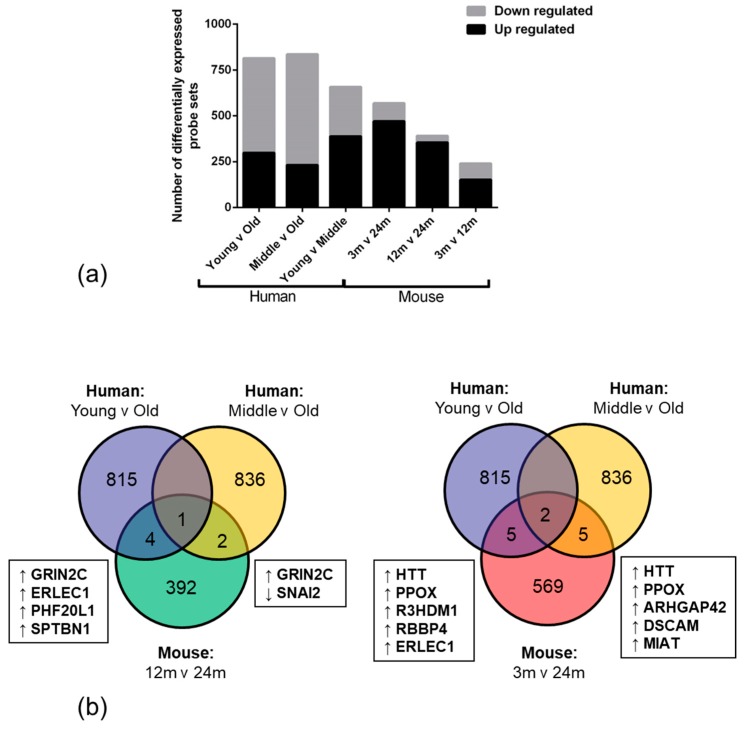
mRNA gene expression analysis. (**a**) Total number of significantly differentially expressed probe sets and the ratio of up to down regulated transcripts in an ageing human and mouse cohort. (**b**) The number of unique and overlapping significantly, differentially expressed genes between the human and mouse datasets. Common genes, dysregulated in the same direction, between species are listed. Up and down regulation compared to oldest age group are represented with directional arrows.

The number of significantly down-regulated genes in the BBB was higher than significantly up-regulated genes in human old age samples when compared to the young (299 up, 516 down, *p* < 0.01) and middle age groups (232 up, 604 down, *p* < 0.01). In contrast, the number of significantly up-regulated genes in the BBB was higher than down-regulated genes in the mouse 24-month age group when compared to either the 3 month (355 up, 37 down, *p* < 0.01) or 12 month time points (471 up, 98 down, *p* < 0.01) (Figure 1a). Analysis of significantly, differentially expressed genes that were altered in the same direction in both mouse and humans with ageing revealed little overlap, with 12 overlapping genes (Rho GTPase-activating protein 42 (*ARHGAP42*), Down syndrome cell adhesion molecule (*DSCAM*), endoplasmic reticulum lectin 1 (*ERLEC1*), glutamate ionotropic receptor NMDA type subunit 2C (*GRIN2C*), huntingtin (*HTT*), myocardial infarction associated transcript (*MIAT*), PHD finger protein 20 like 1 (*PHF20L1*), Snail family transcriptional repressor 2 (*SNAI2*), spectrin β, non-erythrocytic 1 (*SPTBN1*), protoporphyrinogen oxidase (*PPOX*), R3H domain containing 1 (*R3HDM1*) and histone-binding protein RBBP4 (*RBBP4*))(Figure 1b).

### 2.2. Pathway Enrichment Reveals Age-Related Changes in Genes Associated with DNA Binding and Apoptosis/Autophagy

**Figure 2:**
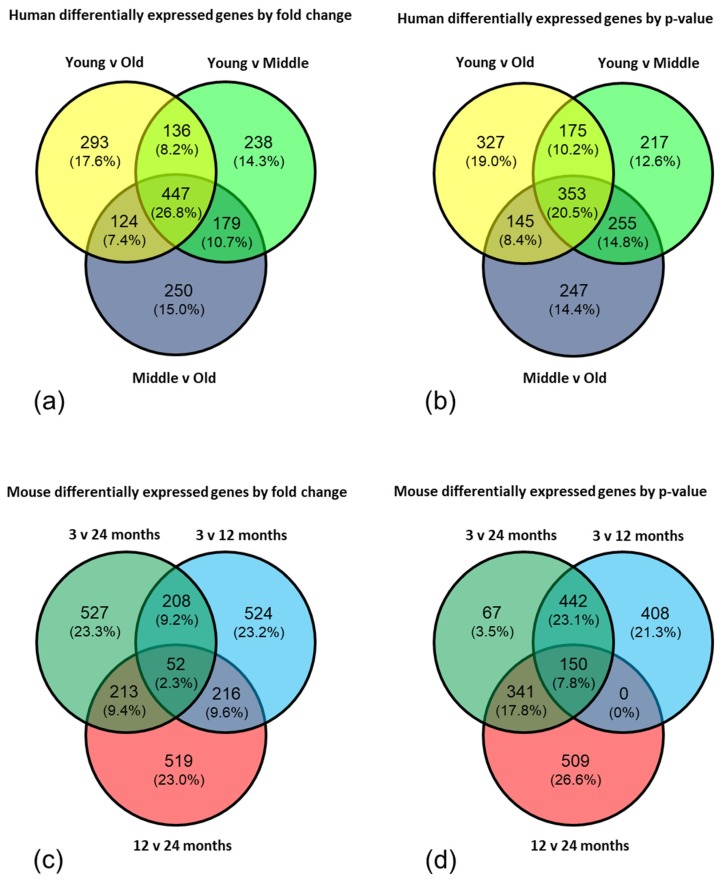
Analysis of top 1000 genes from each comparison in both the human and mouse datasets. In the human dataset, analysis of the genes sorted (**a**) by fold change identified 447 significantly, differentially expressed genes common to all three comparisons, and (**b**) by *p*-value identified 353 genes. In the mouse dataset, analysis of the genes sorted (**c**) by fold change identified 52 significantly, differentially expressed genes common to all three comparisons, and (**d**) by *p*-value identified 150 genes.

Analysis of genes grouped by molecular function optimises false negative and false positive rates [38]; therefore we employed EnrichR and DAVID to interrogate the top 1000 genes from each comparison in both the human and mouse datasets either by fold change or by *p*-value in order to identify common pathways/functional groups affected by ageing. In the human dataset, analysis of the genes sorted by fold change identified 447 significantly, differentially expressed genes common to all three age group comparisons (Figure 2a), and by *p*-value identified 353 genes (Figure 2b). In the mouse dataset, analysis of the genes sorted by fold change identified 52 significantly, differentially expressed genes common to all three comparisons (Figure 2c), and by *p*-value identified 150 genes (Figure 2d).

The overlapping gene lists from each analysis were inputted into DAVID, with the aim of identifying gene networks and clusters of functionally similar genes. In the human dataset, analysis of the overlapping genes common to all three comparisons in the genes sorted by fold change identified three clusters with an enrichment score greater than 1.3 (DNA binding, kelch-like, tRNA modification), and in the genes sorted by *p*-value identified two clusters (DNA binding, apoptosis)(Table 1). In the mouse dataset, analysis of the overlapping genes common to all three comparisons in the genes sorted by *p*-value identified three clusters with an enrichment score greater than 1.3 (GTP binding, Armadillo-like, ion channel binding)(Table 1), but did not identify any clusters in the genes sorted by fold change using a high stringency filter.

To further analyse the data, the 353 human genes and 150 mouse genes from the overlapping genes common to all three comparisons in the genes sorted by *p*-value were assessed using EnrichR. Both the Reactome 2016 database and GO biological process indicated that both lists were significantly enriched for BH3-only proteins, which play an important role in initiating apoptosis and autophagy. Analysis of the human dataset identified: BCL2-like 11 (*BCL2L11*), BCL2 associated agonist of cell death (*BAD*) and tumor protein p53 (*TP53*), *p* = 0.0069 (Reactome 2016) and *p* = 0.0036 (GO biological process). Analysis of the mouse dataset identified: protein phosphatase 3 catalytic subunit gamma (*PPP3CC*), catenin beta 1 (*CTNNB1*), BH3 interacting domain death agonist (*BID*) and karyopherin subunit alpha 1 (*KPNA1*), *p* = 0.0073 (Reactome 2016).

### 2.3. Age-Associated miRNA Expression Changes in the Ageing BBB

**Figure 3:**
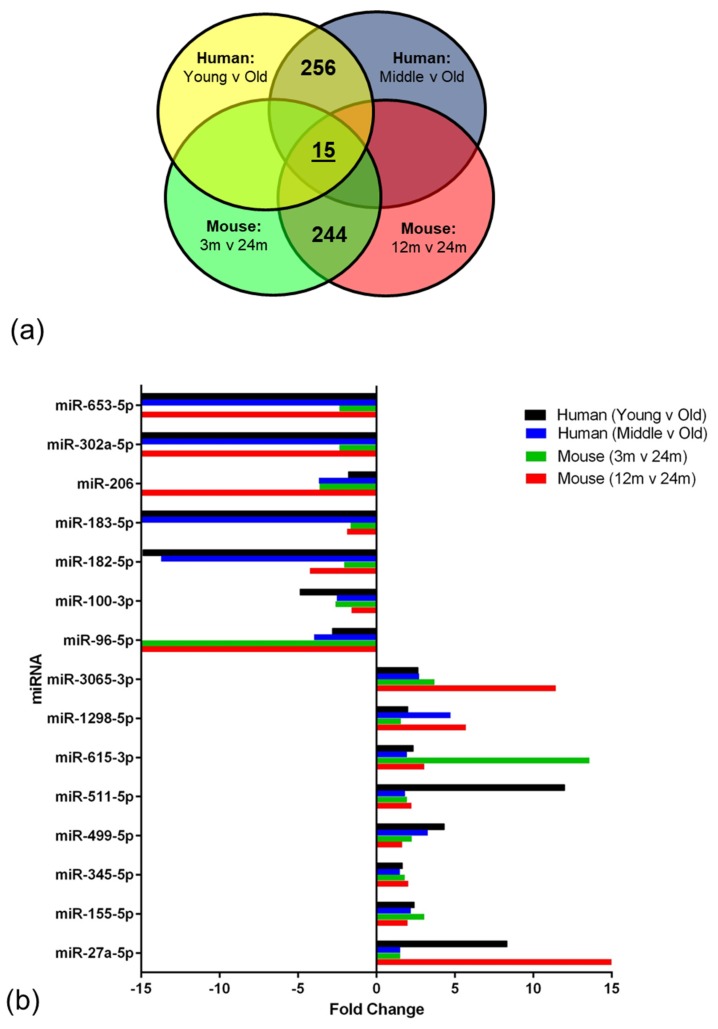
miRNA expression analysis. (**a**) miRNA expression in the human and mouse age group comparisons. Numbers of overlapping miRNAs within species and between all four comparisons are indicated in appropriate sections. (**b**) fold change profiles for the panel of 15 overlapping miRNAs between species.

In the human miRNA data analysis, 256 miRNAs were differentially altered in the same direction when the oldest group was compared to the younger cohort, and in the mouse analysis, 244 miRNAs were altered in the same direction. All significant age-associated miRNA expression changes in the BBB of human and mice are freely available at Gene Expression Omnibus. From these results, 15 miRNAs were identified that overlapped and showed altered expression in the ageing NVU of both species (miR-653-5p, miR-302a-5p, miR-206, miR-183-5p, miR-182-5p, miR-100-3p, miR-96-5p, miR-3065-3p, miR-1298-5p, miR-615-3p, miR-511-5p, miR-499-5p, miR-345-5p, miR-155-5p, miR-27a-5p), as shown in Figure 3.

The Targetscan software was used to specifically identify miRNAs with target genes relating to DNA binding and autophagy (identified in the human mRNA analysis). The miRNAs targeting genes related to autophagy were miR-181c-3p and 505-5p which target *TP53* and miR-505-5p which targets arylsulfatase A (*ARSA*), while those targeting genes related to DNA binding are shown in Table 2.

### 2.4. qPCR and Immunohistochemistry Validation of Age-Associated Candidate Gene Expression Changes

**Figure 4:**
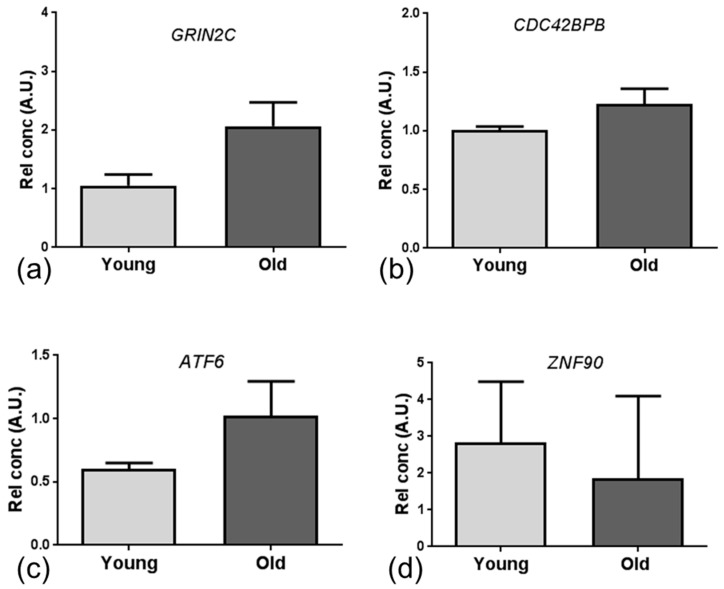
qPCR for mRNA validation of gene expression. A non-significant increase in (**a**) *GRIN2C* (*p* = 0.078), (**b**) *CDC42BPB* (*p* = 0.142), (**c**) *ATF6* (*p* = 0.056) and a non-significant decrease in (**d**) *ZNF90* gene expression was detected in the BBB of the old group, similar to the directional change observed in the microarray analysis. Data presented as mean ± standard error of the mean (SEM).

Validation candidates were selected from the initial microarray analysis based upon species overlap (*GRIN2C* and *SNAI2*), and functional overlap with large fold changes (CDC42 binding protein kinase-β (*CDC42BPB*): fold change (FC) = 5.41). Additional validation candidates were selected from the DNA binding (Activating Transcription Factor 6 (*ATF6*) and zinc finger protein 90 (*ZNF90*)) and apoptosis/autophagy (beclin 1 (*BECN1*), RB1-inducible coiled-coil 1 (*RB1CC1*), tumor protein p53 (*TP53*) clusters identified by the DAVID and EnrichR analyses. Consistent with microarray results, qPCR analysis revealed a similar but non-significant directional change in expression of *GRIN2c* (*p* = 0.078), *CDC42BPB* (*p* = 0.142), *ATF6* (*p* = 0.056) and *ZNF90* (*p* = 0.611) in the ageing human BBB (Figure 4).

**Figure 5:**
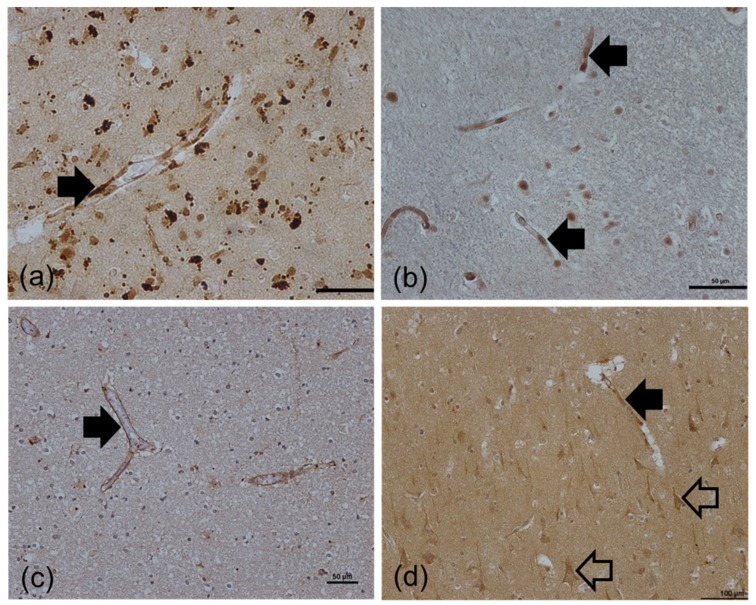
Investigation of protein expression by candidate genes identified in array analysis. (**a**) SLUG (*SNAI2*) and (**b**) p53 (*TP53*) immunolabelled a proportion of endothelial cell nuclei. (**c**) Immunoreactivity for RB1CC1 (*RB1CC1*) was predominantly associated with the basement membrane of capillaries. (**d**) In addition to endothelial immunoreactivity, beclin-1 (*BECN1*) cytoplasmic staining of pyramidal neurones (examples indicated by the open arrow) was present throughout the cortex (Figure 6d). Immunopositive staining of the BBB is indicated by the black arrow. Scale bar represents 50 μm (**a**,**b**,**c**) or 100 μm (**d**).

Immunohistochemistry confirmed expression of the protein encoded by the candidate genes was associated with the BBB. Immunostaining for SNAIL, encoded by *SNAI2*, Figure 5a and p53, encoded by *TP53*, Figure 5b revealed a predominantly nuclear specific signal that was present in a proportion of all cell types, including the BBB where immunoreactive endothelial cell nuclei were identified by their location and typical elongated shape. Immunoreactivity for RB1CC1 was predominantly associated with capillaries as shown in Figure 5c. In addition to endothelial immunoreactivity, beclin-1 cytoplasmic staining of pyramidal neurones was present throughout the cortex, Figure 5d.

**Figure 6:**
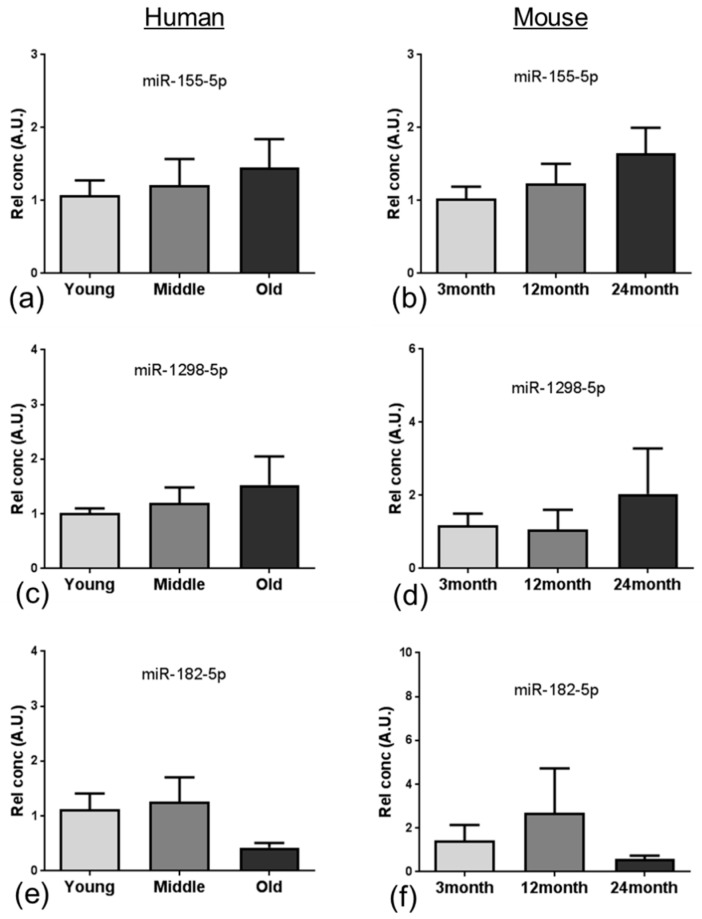
qPCR validation of miRNA expression. qPCR demonstrated the same directional change of expression for all three candidates (miR-155-5p (**a**,**b**); miR-1298-5p (**c**,**d**); miR-182-5p (**e**,**f**)) in both the human (**a**,**c**,**e**) and mouse (**b**,**d**,**f**) groups, similar to the directional change observed in the microarray analysis. Only miR-182-5p in human samples was significantly altered (ANOVA *p* = 0.032). Data presented as mean ± SEM.

qPCR demonstrated the same directional change of expression for miR-155-5p (Figure 6a,b), miR-1298-5p (Figure 6c,d) and miR-182-5p (Figure 6e,f) in both the human and mouse ageing groups, consistent with the directional change observed in the miRNA analysis. Only the age change in miR-182-5p in human samples achieved statistical significance in the oldest group (*p* = 0.032).

## 3. Discussion

Vascular pathology and dysfunction of the NVU may contribute to the onset and progression of AD [39], however the molecular mechanisms underpinning these changes in the ageing brain are poorly defined. Using a combined LCM and expression profiling approach, the current study characterised age-associated changes in the RNA profile of the NVU in an ageing mouse model and an ageing human cohort, identifying mRNA and miRNA expression changes at the NVU which suggest a role for the dysregulation of autophagy and DNA binding in age-associated vascular pathology.

High throughput RNA expression technology enables the use of genome-wide approaches to explore and identify differences in biological conditions, such as ageing. We report that the overall number of significantly down-regulated genes was higher in human old age samples when compared to the young samples. This observation suggests that ageing negatively impacts the transcriptome and may result in an overall age-associated decline in function in the NVU. The high number of upregulated genes in the mouse ageing cohort however, may reflect a developmental expression profile or species difference [40].

Only 12 overlapping genes were altered in the same direction in the NVU of both ageing human and mouse cohorts, including genes which play an important role in regulating vascular tone (*ARHGAP42*) [41]; cell adhesion, neurite outgrowth and axon guidance (*DSCAM*) [42]; repression of tight junction protein expression (*SNAI2*) [43,44]; cellular stress-response (*ERLEC1*) [45]; learning, memory and synaptic development (*GRIN2C*) [46], all processes prone to dysregulation with advancing age.

Given the low number of overlapping specific candidate genes, we employed integrated mRNA and miRNA network and pathway enrichment analysis of the ageing mouse and human NVU datasets, and identified transcriptomic changes associated with DNA binding and apoptosis/autophagy pathways. The major miRNA-targeted genes associated with DNA binding included zinc finger proteins (*ZNF*), activating transcription factor (*ATF*) and the Forkhead family of transcription factors (*FOX*). ZNF have a wide range of functions including interacting with DNA to regulate transcription, as reviewed in [47]. In the current study we identified dysregulated expression of several members of the ZNF family, including *ZNF704*, which is a suggested potential candidate gene for healthy ageing [48], and *ATF6*, a member of the leucine zipper family of transcription factors which plays a major role in regulating tissue homeostasis in response to stress [49], and is expressed at high levels in a range of neurodegenerative diseases including amyotrophic lateral sclerosis [50]. We also report dysregulated expression of several members of the FOX transcription factors, including *FOXA1* which plays a role in the response to stress and is associated with the AD-gene signature [51] and *FOXP1* which regulates expression of immune genes [52]. Together these findings suggest that dysregulation of DNA binding genes which regulate transcription of stress response and immune response-related genes may be an early event in age-associated changes at the NVU.

Several studies indicate dysregulation of autophagy, a lysosome-dependent process in which organelles and proteins are degraded, as a contributing factor to the pathogenesis of AD, as recently reviewed [53], with autophagy-associated markers detected in vessels in AD [54] and impaired autophagic protein degradation associated with apoptosis of endothelial cells [55]. In addition to identifying dysregulation of autophagy-associated *BECN1* and the apoptosis-related gene *TP53*, we also confirmed endothelial expression of beclin-1 and p53 by endothelial cells, supporting the findings of previous studies [56,57], and suggesting that dysregulation of the apoptosis/autophagy pathway may contribute to dysfunction of the NVU in the ageing brain.

miRNAs regulate a diverse range of biological processes by interacting with their target mRNA, usually to repress translation [58]. In the current study, integrated analysis of miRNA and mRNA expression profiling identified 15 miRNAs that overlapped and showed altered expression in the ageing NVU of both species. In addition to targeting genes related to DNA binding and/or autophagy, many of these well characterised miRNAs play a role in several age-relevant processes. We report increased expression of miR-155, one of the most well characterised miRNAs, which has also been detected in patients and animal models of AD [59] and Down syndrome dementia [60,61], and has been shown to play a role in regulating the neuroinflammatory response in AD [62] and contributing to BBB dysfunction in multiple sclerosis [63] and cerebral malaria [64]. While the majority of miR-345 studies to date have focussed on the role in the induction of apoptosis in cancer [65,66], miR-345-5p has been identified as a potential blood biomarker in multiple sclerosis patients [67], indicating that this miRNA plays a role in neurological disease. Interestingly miR-27a has recently been shown to protect against BBB dysfunction in a mouse model of intracerebral haemorrhage [68] and to protect against traumatic brain injury by suppressing autophagy [69], suggesting that the significant increased expression of miR-27a in the ageing NVU may play a neuroprotective role.

Several of the miRNAs identified in the analysis presented a significant reduction in expression, including miR-96, which is associated with an increased neuroinflammatory response [70]; miR-100 which regulates expression of major components of the mechanistic target of rapamycin (mTOR), transforming growth factor-β and insulin signalling pathways in brain endothelial cells [71]; miR-182 and miR-183 which regulate SUMOylation and act to preserve homeostasis under stress [72]; and miR-206, which is differentially expressed in patients with mild cognitive impairment, as recently reviewed [73].

While our study discusses our findings with respect to ageing, it should be acknowledged that other factors may have influenced BBB dysfunction in the human cohort. For example, raised venous pressure peri-mortem may have impacted the BBB in the youngest age group cases that died by suspension ligature [74], and ischemic heart disease may have impacted the BBB in the older cohort [75]. Such co-morbidities are an inevitable limitation with human autopsy cohorts.

Animal models of ageing and age-associated disease are crucial to research and offer the opportunity to identify novel molecular mechanisms underlying age-associated neuropathology, however, the findings of the current study suggest that the data generated in these models should be interpreted with caution as not all the candidates identified are relevant to human disease and cannot be directly translated. Overall our integrated mRNA and miRNA network analysis of the NVU transcriptome in ageing human and mouse cohorts identified dysregulation of apoptosis/autophagy and DNA binding networks, in addition to biologically relevant RNA changes which impact BBB function and CNS homeostasis. Future studies to interrogate these candidate changes in more detail have the potential to develop targeted therapeutic approaches to prevent vascular dysfunction in the ageing brain.

## 4. Materials and Methods

### 4.1. Study Cohort

Frozen samples of post mortem human prefrontal association cortex (Brodmann areas 8/9) from cases without a history of neurological disease were obtained from the Edinburgh Medical Research Council Sudden Death Brain Bank, who granted approval for the use of tissue in this study (Edinburgh Brain Bank REC reference 11/ES/0022). The cohort represented young adult (20–30 years [y], *n* = 5), middle aged (44–57 y, *n* = 5) and old aged individuals (71–79 y, *n* = 5). Haematoxylin and eosin stained sections were examined from each case by a neuropathologist (S.B.W.), and immunohistochemistry was carried out with antibodies phospho-tau (AT8) and β-amyloid, to document age-associated pathological changes (Table 3).

C67BL/6 male mice were purchased from Charles River at 3, 12 and 24 months old (*n* = 5 per group). Animals were sacrificed, perfused with phosphate buffered saline (PBS) and the brain dissected and snap frozen in liquid nitrogen. All animal experiments were conducted following ethical review processes in accordance with the Animals (Scientific Procedures) Act 1986 of the UK government (Home Office Project Licence Number 8002612, approval date 30th April 2013) and the ARRIVE guidelines (Animal Research: Reporting of In Vivo Experiments: https://www.nc3rs.org.uk/arrive-guidelines).

### 4.2. Laser Capture Microdissection

**Figure 7:**
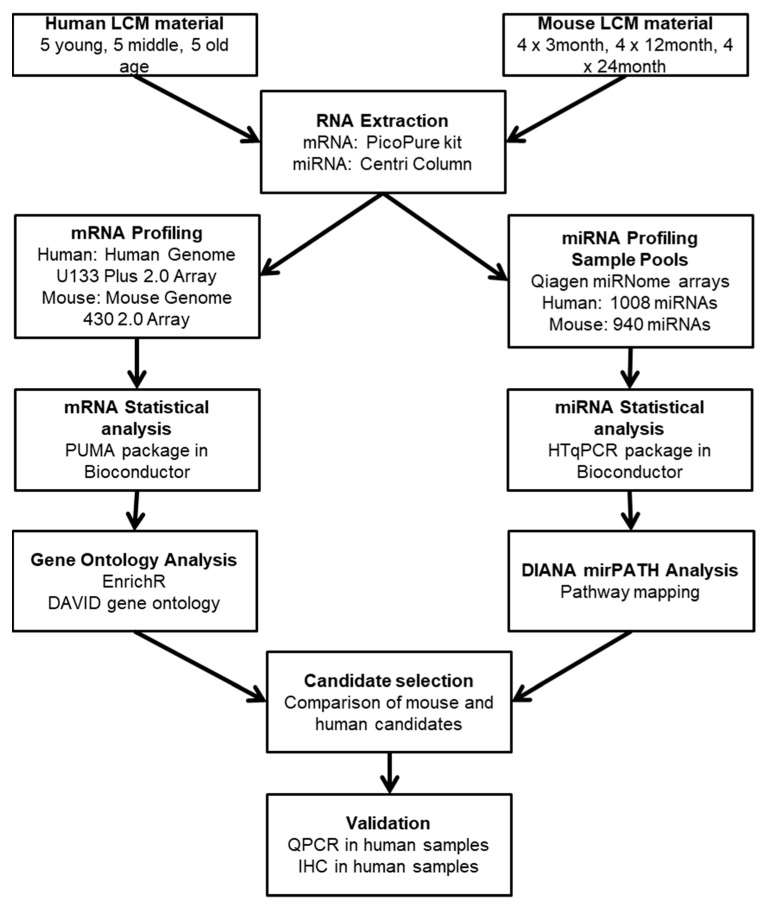
Workflow of the study design, including initial mRNA and miRNA expression profiling, bioinformatic analysis and validation. LCM: laser capture microdissection. IHC: immunohistochemistry. qPCR: quantitative polymerase chain reaction.

Laser capture microdissection (LCM) of microvascular cells was performed using a standard protocol [76]. Frozen sections (7 μm) were stained for Collagen IV (1:200, AbCam, UK ab6586) using a rapid avidin-biotinylated complex-horse radish peroxidase complex (ABC-HRP) immunostaining protocol to visualise vessels. Subsequently, sections were dehydrated in a graded series of ethanol, extensively cleared in xylene and air dried for 1 h. Microvascular cells were microdissected using an Arcturus Veritas Laser Capture Microdissection System (Arcturus Bioscience Inc., Mountainview, CA, USA), and collected onto two thermoplastic-coated CapSure caps (ThermoFisher, Altrincham UK) per sample. Following microdissection, caps were secured in 0.5 mL tubes, incubated with RNA PicoPure^®^ extraction buffer (Life Technologies, Paisley, UK) at 42 °C for 30 min. A work flow of the study design is outlined in Figure 7.

### 4.3. Total RNA Extraction

The cell/extraction buffer solution from two caps per sample was combined, then divided equally. For mRNA extraction, 50 μL of sample was taken through the PicoPure^®^ RNA isolation protocol according to the manufacturer’s instructions, with mRNA eluted in 10 μL of PicoPure^®^ elution buffer. For miRNA extraction, the remaining 50 μL of sample was passed through a Centri Spin™-10 purification column (Princeton Separations Inc, Adelphia, NJ, USA) according to manufacturer’s instructions. All RNA samples were stored at −80 °C prior to expression profiling.

### 4.4. mRNA Expression Profiling

Transcriptional profiling of the BBB isolated from an ageing mouse cohort was performed using Affymetrix Mouse Genome 430 2.0 Arrays (Affymetrix, Santa Clara, CA, USA). In the human cohort we profiled samples using Human Genome U133 PLUS 2.0 Arrays (Affymetrix). Total RNA was annealed to an oligo-d(T) primer with a T7 polymerase binding site. After generation of double-stranded cDNA, copy RNA (cRNA) was transcribed which then formed the RNA template for a second round of amplification. At the end of this round, after synthesis of double-stranded cDNA, biotin-labelled cRNA was prepared using the Affymetrix Gene Chip (Affymetrix) in vitro transcription labelling kit. Following clean-up of the biotin-labelled cRNA the material was assayed (Agilent Bioanalyser 2100, Stockport, UK) to ensure sufficient RNA of appropriate quality had been prepared. Labelled cRNA (12.5 µg) was fragmented, applied to the gene chips and hybridised over 16 h at 45 °C in a rotating oven at 60 rpm. Post hybridisation washing and sample staining was carried out using the Fluidics Station 400 and the Gene Chip Operating System (GCOS). Gene chips were scanned using the GC3000 7G scanner and data processed for quality control using Expression Console software (Affymetrix) and analysis carried out using Qlucore Omics Explorer (Qlucore, Lund, Sweden). Further analysis was carried out using R statistical language as detailed below.

### 4.5. miRNA Expression Profiling

Extracted miRNA samples from each age group (3, 12 and 24 months in the mice and young, middle, old in humans) were pooled. miRNA profiling was performed with miRNome miScript miRNA PCR arrays (Qiagen, Hilden, Germany) in accord with the manufacturer’s recommendations, using a CFX Real-Time PCR System instrument (Bio-Rad, Hercules, CA, USA). All miRNome qPCR experiments were performed in 384 well plates, with a set of normalisation and technical controls repeated on each plate.

### 4.6. Data Analysis: mRNA

Analysis of microarray output was conducted using the R statistical language with statistical packages available from Bioconductor (LIMMA and PUMA) (www.bioconductor.org). Multi-chip modified gamma model for oligonucleotide signal (MMGMOS) normalization, using median global scaling, followed by present/absent MAS5 filtering, was applied to process the data. Subsequently, the improved probability of positive log ratio (IPPLR) in PUMA was used to identify differentially expressed genes between the age groups in each species, minimum 1.2 fold change (FC). In the human data, the middle aged group comprised of exclusively male samples, to eliminate potential influence of gender bias in the data a PUMA comparison of male versus female genes was completed within the old age group, and significantly differentiated genes removed from further age group comparisons. The gene lists were analysed by EnrichR and the Database for Annotation Visualisation and Integrated Discovery bioinformatics programme (DAVID) was used to group genes according to their function [77,78].

To further interrogate the data, the top 1000 genes from each comparison (young versus old; young versus middle aged; middle aged versus old) in both the human and mouse datasets were selected by fold change and by *p*-value and Venn diagrams created to highlight the significantly, differentially expressed genes which appeared in all three comparisons. The gene lists from the centre of each Venn diagram were analysed by EnrichR and DAVID using a high stringency filter to reduce the rate of false positives, and an enrichment score >1.3 (corresponding to a *p*-value <0.05) considered significant.

### 4.7. Data Analysis: miRNA

Expression data for 1008 human miRNAs and 940 mouse miRNAs obtained from the Qiagen miRNome PCR arrays was analysed using the HTqPCR package from Bioconductor [79]. Data was filtered to remove miRNAs with a *C*t value >37 in all samples. Geometric mean normalisation was applied to each individual 384 well plate, followed by global normalisation using the quantile method after data was combined to constitute each miRNome dataset. NormFinder analysis of internal controls was used to identify the most stable genes (Human: *SNORD61* and *SNORD68*, mouse: *SNORD68* and *SNORD96A*) for Δ*C*t and fold change calculations using the ΔΔ*C*t method of relative quantification. Subsequently, miRNAs were ranked according to fold change in age group comparisons and considered differentially expression based on ≥1.5 fold change. Enrichment analysis using Diana mir-Path software allowed identification of molecular pathways potentially altered by the expression of multiple miRNAs. Target genes of these miRNAs were downloaded from Targetscan and compared to the mRNA array datasets.

### 4.8. Quantitative PCR

RNA was extracted from laser captured microvessels using PicoPure RNA isolation kit as described above and cDNA synthesized with the qScript cDNA supermix kit (Quanta Biosciences, Gaithersberg, MD, USA) in a G-Storm thermocycler (G-Storm, Somerton, UK). In contrast to the miRNA array profiling which was conducted on pooled samples from each age group, for qPCR individual samples in each age group were analysed. qPCR was performed using IDT PrimeTime qPCR assays (Integrated DNA Technologies, Glasgow, UK) and Brilliant qPCR mix (Agilent) in a reaction volume of 5 μL using a CFX Real-Time PCR System instrument (Bio-Rad) (Table 4). β-actin was amplified on each plate to normalize expression levels of target genes using the ΔΔCt method, and differences in mRNA or miRNA expression assessed by student’s t-test or ANOVA, respectively.

### 4.9. Immunohistochemistry

To confirm BBB expression of proteins encoded by the candidate genes, immunostaining was performed using a standard avidin biotinylated enzyme complex (ABC) method, and the signal visualised with diaminobenzidine (Vector Laboratories, Peterborough, UK). A summary of the primary antibodies used is shown in Table 5.

## Figures and Tables

**Table 1:** Gene network and cluster analysis of differentially expressed genes common to all three comparisons (young versus old; young versus middle aged; middle aged versus old) in both the human and mouse datasets, selected by fold change and by *p*-value.

Dataset	Cluster	No. Genes	Enrichment Score
Human (sorted by FC)	DNA binding	6	1.88
	Kelch-like	5	1.73
	tRNA modification	4	1.46
Human (sorted by *p*-value)	DNA binding	5	1.45
	Apoptosis	3	1.32
Mouse (sorted by *p*-value)	GTP binding	7	2.32
	Armadillo-like	4	1.81
	Ion channel binding	3	1.76

FC, fold change.

**Table 2:** miRNAs which target genes relating to DNA binding. The shaded box indicates that a gene is present in the list of targets of the miRNA. This comparison has a highly significant *p*-value of 1 × 10^−12^. All but two of the genes present in the list are targets of more than one miRNA.

miRNA Gene Target	1298-5p	653-5p	511-3p	505-5p	345-5p	302a-5p	224-5p	205-5p	182-5p	181c-3p	155-5p	100-3p	27a-5p	Total
*NFIB*														9
*ZNF704*														7
*EBF1*														6
*FOXP1*														6
*IKZF1*														5
*ZFHX4*														4
*FLI1*														4
*ATF6*														4
*HNRNPD*														4
*MAFF*														4
*RCOR3*														3
*ZKSCAN3*														3
*ZNF514*														3
*FOXA1*														2
*ZNF268*														2
*ZNF90*														2
*TP53*														2
*RAD51D*														2
*ERCC1*														2
*ZNF600*														2
*FOXR2*														1
*TCF7L1*														1

**Table 3:** Demographics of human cases used in gene expression analysis: age, gender, post-mortem delay, brain pH, cause of death and neuropathology.

Age Group	Age	Gender	PMD	pH	Cause of Death	Neuropathology
**Young**	20	F	71	6.5	SBL	None
	24	F	47	6.4	SBL	Small vessel disease
	25	M	53	6.4	SBL	None
	29	M	44	6.5	SBL	Focal Tau
	30	M	71	6.4	NK	None
**Middle aged**	44	M	47	6.3	Drug overdose	None
	48	M	72	6.3	CAD	None
	50	M	45	6.3	IHD/CAD	None
	52	M	91	6.4	Road traffic collision	None
	57	M	66	6.5	IHD/CAD	None
**Old**	71	F	41	6.5	IHD	Mild and focal vascular tau. Mild amyloid tangles and plaques
	74	M	46	6.3	Pulmonary thromboembolism	None
	74	M	66	6.3	IHD/CAD	Mild tau tangles, plaques and threads.
	75	M	78	6.4	IHD/CAD	Mild tau tangles and threads
	79	F	45	6.3	IHD/CAD	Venous collagenosis and small vessel disease

Abbreviations: PMD: post-mortem delay in hours; M: male; F: female; SBL: suspension by ligature; NK: not known; IHD: ischaemic heart disease; CAD: coronary artery disease.

**Table 4:** qPCR primer/probe sequence.

Gene		Sequence
*ATF6*	Probe	5′-FAM/CATTCCTCCACCTCCTTGTCAGCC-3′
	Primer 1	5′-CTTGGTCCTTTCTACTTCATGTCT-3′
	Primer 2	5′-CCCTGATGGTGCTAACTGAA-3′
*CDC42BPB*	Probe	5′FAM/ACAAAGAGCCTGATTCGGACTCCAC-3′
	Primer 1	5′-GGAGCTATTCGATGGAGTTGAG-3′
	Primer 2	5′-GAACAAGCCCTACATCTCGTG-3′
*GRIN2C*	Probe	5′-FAM/ AAGGCATCCAGCTTCCCCATCTT-3′
	Primer 1	5′-TTGAGGA -3′AGCAGCATCATAG-3′
	Primer 2	5′-CGCAGTAACTACCGTGACAT-3′
*ZNF90*	Probe	5′-FAM/AGCTGTGGATCTCCCAATACCTGC-3′
	Primer 1	5′-GGCCACATCTCTAAATTCCAATG-3′
	Primer 2	5′-CTTAGCTGCTTCGTGTCTTCT-3′
*ACTB*	Probe	5′-FAM-CCATGTACGTTGCTATCCAGGCTGT-3′
	Primer 1	5′-CCAGTGGTACGGCCAGA-3′
	Primer 2	5′-GCGAGAAGATGACCCAGAT-3′

**Table 5:** Antibody source and specificity.

Antibody	Isotype	Dilution	Antigen Retrieval	Supplier
Beclin-1	Rabbit IgG	1:250 (60 min, RT)	PC EDTA	AbCam
p53	Mouse IgG	1:50 (o/n, 4 °C)	PC EDTA	Santa Cruz
RB1CC1	Rabbit IgG	1:100 (60 min, RT)	PC TSC	Sigma Aldrich
SLUG	Rabbit IgG	1:200 (60 min, RT)	MW TSC	AbCam

Abbreviations: RT: room temperature; o/n: overnight; PC: pressure cooker (125 °C for 30 s at 20 psi); MW: microwave; EDTA: ethylenediaminetetraacetic acid (pH 8); TSC: trisodium citrate (pH 6.5).

## Abbreviations

ABC-HRP	Avidin-biotinylated complex-horse radish peroxidase
AD	Alzheimer’s disease
ARHGAP42	Rho GTPase-activating protein 42
ARSA	arylsufatase A
ATF	Activating Transcription Factor
BAD	BCL2 associated agonist of cell death
BBB	blood-brain barrier
BCL2L11	BCL2-like 11
BECN1	beclin 1
BID	BH3 interacting domain death agonist
CDC42BPB	CDC42 binding protein kinase-β
CNS	central nervous system
CSF	cerebrospinal fluid
CTNNB1	catenin beta 1
DAVID	Database for Annotation Visualisation and Integrated Discovery
DSCAM	Down syndrome cell adhesion molecule
EBF1	early B-Cell Factor 1
ERCC1	excision repair cross-complementation group 1
ERLEC1	endoplasmic reticulum lectin 1
FC	fold change
FLI1	friend leukemia integration 1 transcription facto
FOX	Forkhead family of transcription factors
GEO	Gene Expression Omnibus
GFAP	glial fibrillary acidic protein
GRIN2C	glutamate ionotropic receptor NMDA type subunit 2C
HTT	huntingtin
IKZF1	Ikaros family zinc finger protein 1
IPPLR	improved probability of positive log ratio
KPNA1	karyopherin subunit alpha 1
LCM	laser capture microdissection
MAFF	v-maf avian musculoaponeurotic fibrosarcoma oncogene homolog F
MIAT	myocardial infarction associated transcript
miRNA	microRNA
MMGMOS	multi-chip modified gamma model for oligonucleotide signal
mRNA	messenger RNA
mTOR	mechanistic target of rapamycin
NFIB	nuclear factor I B
NFL	neurofilament light
NVU	neurovascular unit
PBS	phosphate buffered saline
PHF20L1	PHD finger protein 20 like 1
PPOX	protoporphyrinogen oxidase
PPP3CC	protein phosphatase 3 catalytic subunit gamma
R3HDM1	R3H domain containing 1
RBBP4	histone-binding protein RBBP4
RB1CC1	RB1-inducible coiled-coil 1
RCOR3	REST Corepressor 3
SNAI2	Snail family transcriptional repressor 2
SPTBN1	spectrin β, non-erythrocytic 1
TCF7L1	transcription factor 7-like 1
TP53	tumor protein p53
vWF	von Willibrand factor
ZFHX4	zinc finger homeobox 4
ZKSCAN3	zinc finger with KRAB and SCAN domains 3
ZNF	zinc finger protein
